# Concurrent Systemic Lupus Erythematosus and Hashimoto's Thyroiditis: Clinical Patterns From Two Cases in Southwestern Nigeria

**DOI:** 10.1155/crie/6623717

**Published:** 2025-11-04

**Authors:** Gbenga Joshua Odunlami, Tajudin Adesegun Adetunji, Bolanle Aderonke Omotoso, Meveilleoux Soronuchi Frankpeace, Adeyemi Abiola Adefidipe, Francis Sunday Igwe, Airenakho Emorinken

**Affiliations:** ^1^Department of Medicine, Obafemi Awolowo University, Ile-Ife, Osun, Nigeria; ^2^Department of Medical Pharmacology and Therapeutics, Obafemi Awolowo University, Ile-Ife, Osun, Nigeria; ^3^Department of Medicine, Obafemi Awolowo University Teaching Hospitals Complex, Ile-Ife, Osun, Nigeria; ^4^Department of Morbid Anatomy and Forensic Medicine, Obafemi Awolowo University Teaching Hospitals Complex, Ile-Ife, Osun, Nigeria; ^5^Department of Radiology, Obafemi Awolowo University Teaching Hospitals Complex, Ile-Ife, Osun, Nigeria; ^6^Department of Medicine, Irrua Specialist Teaching Hospital, Irrua, Edo, Nigeria

## Abstract

Systemic lupus erythematosus (SLE) is an autoimmune, multisystemic connective tissue disease with a predilection for women in their reproductive age group. Hashimoto's thyroiditis (HT) is a chronic autoimmune-mediated inflammation of the thyroid gland. The coexistence of SLE with HT has been reported in the literature. However, this important coexistence has scarcely been reported in sub-Saharan Africa. Hence, we report two patients who presented with HT coexisting with SLE.

## 1. Background

Systemic lupus erythematosus (SLE) is a multisystemic autoimmune connective tissue disease that is characterised by the elaboration of autoantibodies against the nuclear or cytoplasmic antigens, remissions and flares, and variable clinical course and prognosis [1]. It is common in women in their reproductive years [2]. It is much more common in black Africa than was previously reported [3].

Hashimoto's thyroiditis (HT) is a chronic autoimmune disease affecting the thyroid gland and is characterised by lymphocytic infiltration of the thyroid gland and destruction of the thyroid follicular cells. It can occur in all age groups and is 5–20 times more common in women than men. It is reported to be common in Asians and Caucasians than in blacks [4]. A study by Posselt et al. [5] showed that the prevalence of HT was twice as high among SLE patients compared to controls. However, the coexistence of HT with SLE has not yet been reported in sub-Saharan Africa. Hence, a report of these two cases aims to raise awareness of this important occurrence in our environment.

### 1.1. Case Report

The first case is a 67-year-old woman who presented to the nephrology clinic 13 years ago on account of progressive anterior neck swelling, fatigue, cold intolerance, severe recurrent joint pains, and swelling involving the wrists and hand joints, weight loss, and painless oral ulcers. She was also diagnosed as hypertensive on presentation. Laboratory investigations revealed anaemia, thrombocytopenia, elevated erythrocyte sedimentation rate (ESR), elevated titres of antinuclear antibody (ANA) with a speckled immunofluorescence pattern, and anti-double-stranded deoxyribonucleic acid antibody (dsDNA). Thyroid function tests at presentation revealed low free tri-iodothyronine (FT3), low free thyroxine (FT4), high thyroid-stimulating hormone (TSH), and elevated thyroid peroxidase (anti-TPO) and anti-thyroglobulin antibodies. Her fasting lipid profile was also deranged (Table 1). A neck ultrasound showed an enlarged thyroid with multiple nodules, especially in the isthmus. There was no evidence of renal or other manifestations of SLE. Diagnosis of SLE was made using the 2019 American College of Rheumatology/European League of Associations for Rheumatology (ACR/EULAR) criteria in coexistence with HT. Using the criteria, she had a cumulative score of 18, based on thrombocytopenia, oral ulcers, arthritis, and anti-dsDNA, exceeding the minimum score of 10 needed to be met for diagnosis. She was commenced on oral levothyroxine, prednisolone, hydroxychloroquine, atorvastatin, amlodipine, and lisinopril. She responded well to the treatment over the years and has been in remission till her last clinic visit. Her blood pressure has also been well-controlled.

**Figure 1:**
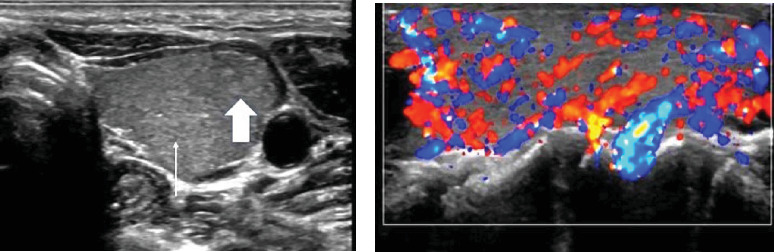
B-mode (a) and Duplex (b) ultrasonographic images of the thyroid gland of the 23-year-old patient, showing coarsened parenchyma echotexture (thin arrow) and multiple hyperechoic nodules consistent with pseudonodular appearance (thick arrow) on B-mode, while on Doppler ultrasound, it shows hypervascularity. These appearances are highly specific for Hashimoto thyroiditis.

The second case is a 23-year-old lady who presented to the endocrinology clinic of the Obafemi Awolowo University Teaching Hospitals Complex, Ile-Ife, in July 2023 on account of a 10-month history of painless progressive anterior neck swelling with associated dysphagia and odynophagia. There was also an associated history of slow mentation, lethargy, fatigue, weight gain, cold intolerance, constipation, and menorrhagia. There was no history of neck irradiation or surgery. She subsequently developed inflammatory pain in her elbows and passage of frothy urine with associated early morning facial puffiness 4 months before presentation. Examination of the neck showed anterior neck swelling, smooth, and diffuse, measuring 17 cm by 12 cm. It moves with deglutition but not with tongue protrusion. It was soft with differential warmth. There were no pigmented skin changes or bruit over the mass. Thyroid function tests showed raised TSH, and low free FT4 but normal free FT3. Anti-thyroid peroxidase antibody (TPOAb) was markedly elevated (Table 2). A neck ultrasound was done and revealed the thyroid lobes to be diffusely enlarged with a heterogeneous parenchymal echo pattern and multiple hyper-echoic micronodules diffusely distributed within them. Colour Doppler interrogation demonstrated a high vascular flow (giving the appearance of a thyroid inferno) in both thyroid lobes (Figure 1). All these features were in keeping with HT.

She was commenced on oral levothyroxine 50 mcg/day and subsequently referred to the rheumatology clinic for further review. There was no history of photosensitive skin rash, alopecia, mouth or throat sores, and pleuritic or pericarditic pain. She was not a known hypertensive or diabetic. A general physical examination revealed pallor, supraclavicular, and axillary lymphadenopathy. Other systemic examinations were normal. Electrolytes, urea, creatinine, and serum albumin were also normal. The haematogram showed anaemia, leucopenia, and raised ESR. Urinalysis showed proteinuria (2+) and haematuria (2+). Urine microscopy showed 28–30 red blood cells/high power field. The 24-h urinary protein was in the nephrotic range, and her fasting lipid profile was deranged. ANA titre was 1:2560 with a speckled immunofluorescence pattern. Anti-double-stranded antibody and anti-Smith antibody (anti-Sm) were both elevated (Table 2).

**Figure 2:**
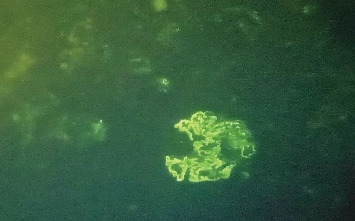
The immunofluorescence imaging of the renal biopsy specimen showing thickening of the capillary basement membrane with a full-house pattern, predominantly along the subepithelial and mesangial areas of the glomerular capillary walls.

Based on the 2019 ACR/EULAR criteria, she had a cumulative score of 23 based on arthritis, leucopenia, Class V lupus nephritis, and anti-dsDNA. Therefore, a diagnosis of SLE was made in addition to the previously diagnosed HT. She was commenced on oral prednisolone 50 mg daily, oral rabeprazole 20 mg daily, oral hydroxychloroquine 200 mg twice daily, oral calcium/vitamin D supplementation, haematinics, oral atorvastatin 10 mg daily, and oral lisinopril 5 mg daily. Renal biopsy was suggestive of Class V lupus nephritis (Figure 2). She was commenced on mycophenolate mofetil 1 g twice daily. She experienced a drastic improvement in her symptoms and laboratory features within 2 months of commencing treatment, and she is currently clinically stable (Table 2).

## 2. Discussion

The coexistence of autoimmune thyroid disease with SLE has been reported in American, European, Asian, and North African studies, but none of them included sub-Saharan Africa [6]. Several studies across wide geographical locations have been done to determine the association between SLE and thyroid autoimmunity, but the results were conflicting, perhaps because some of these studies have relatively small sample sizes, which lacked the adequate power to show any true association. However, a meta-analysis of these studies by Pan et al. [6] established the association between thyroid autoimmunity and SLE. It is still a subject of debate whether SLE is an independent risk factor for autoimmune thyroid disease or a mere coincidence, as both diseases have a predilection for young to middle-aged women [7]. Nevertheless, since both are autoimmune diseases, they may share some common autoimmune features explaining their coexistence [6]. This is evidenced by a retrospective cohort study by Lin et al. [8], which suggests that women with HT have a higher risk of developing SLE. Furthermore, there is emerging evidence to suggest a genetic basis for the association between SLE and autoimmune thyroid disease. Individuals with R620 polymorphism in the protein tyrosine phosphatase (PTPN22) gene encoding a T-cell protein are more likely to develop concurrent SLE and thyroid disease [9]. In another study, a locus on chromosome 5 (5q14.3–15) was found to have a susceptibility gene shared by SLE and autoimmune thyroid disease [10]. Apart from the genetic and immunological factors contributing to autoimmune thyroid disease in SLE, environmental factors have also been implicated. These include alcohol intake, smoking cessation, increased iodine intake, medications (e.g., interferon alpha, lithium), and bacterial and viral infections (e.g., hepatitis C, Yersinia enterolitica) [4, 11–13]. Autoimmune thyroid disease may precede the onset of SLE, simultaneously, or after the diagnosis of SLE. Autoantibodies—particularly anti-TPO and anti-thyroglobulin (anti-Tg)—often appear several years before the development of biochemical or clinical hypothyroidism in HT. Longitudinal studies demonstrate that antibody positivity may be detectable up to 5–7 years prior to diagnosis, with many individuals remaining clinically euthyroid during this latent phase [14]. Patients may pass through a transient thyrotoxic stage (Hashitoxicosis) before entering a euthyroid or subclinical hypothyroid state and ultimately progressing to overt hypothyroidism [15]. HT typically presents with a painless goitre, hypothyroidism, and thyroid antibodies, particularly TPOAb, which has a specificity of 90%–95% for the condition [4]. A thyroid ultrasound is not usually needed but may show an enlarged thyroid gland with a coarse or inhomogeneous echotexture and multiple ill-defined nodules [11].

A number of features of SLE have been associated with autoimmune thyroiditis. These include major organ manifestations (e.g., lupus nephritis, neuropsychiatric lupus), dyslipidemia, anti-dsDNA, anti-Sm antibodies, and high disease activity [16, 17]. Studies have interestingly suggested that the prognosis of both conditions is interrelated, and effective treatment of one may positively impact the outcome of the other [17, 18].

Pericarditis is one of the manifestations of serositis, a common feature of SLE. According to a recent multicentre national study in Nigeria, serositis was present in 32.6% of SLE cases [19]. The reported prevalence of pericarditis in various studies ranges from 11% to 54%. Identified risk factors include male sex, younger age at onset, presence of proteinuria, lymph node enlargement, leucopenia, thrombocytopenia, low complement levels, and positivity for anti-dsDNA and anti-Sm antibodies. In lupus-related pericarditis, true inflammation occurs due to immune complex deposition and complement activation, often resulting in fibrinous or serofibrinous pericardial involvement. Clinical presentations may include acute pericarditis, exudative pericardial effusion, or, in severe cases, cardiac tamponade. Management typically involves anti-inflammatory drugs and immunosuppressive therapy [20]. In contrast, pericardial effusion associated with hypothyroidism occurs in about 3%–37% of cases. This is due to increased capillary permeability and reduced lymphatic clearance of albumin caused by low levels of thyroid hormones. The resulting pericardial effusion is transudative, not immune-mediated, and usually asymptomatic, although cardiac tamponade can occasionally occur. Treatment consists of thyroid hormone replacement [21]. Our reported cases did not present with pericardial effusion.

Though there is a paucity of data on the coexistence between SLE and HT in African populations, this occurrence may be shaped by a combination of genetic susceptibility, environmental triggers, and epidemiological realities. Genetic factors such as APOL1 risk variants, which are prevalent in individuals of African ancestry and linked to renal manifestations of SLE, may reflect broader immune dysregulation with potential relevance to thyroid autoimmunity. Epidemiologically, while the true burden of SLE in sub-Saharan Africa has been historically underestimated due to limited diagnostic resources and healthcare access, meta-analyses now indicate a substantial and under-recognised prevalence—ranging broadly but with early onset particularly in young women—creating an environment conducive to autoimmune clustering [22, 23]. Environmentally, exposures such as air pollution and other agents that induce epigenetic dysregulation, notably DNA hypomethylation in immune cells, which also contributes to increased IFN-α in blacks, have been implicated in the pathogenesis of SLE and may extend to thyroid autoimmunity via shared autoimmunity-promoting mechanisms [24].

A diagnosis of multiple autoimmune syndrome (MAS) type 3 includes HT, a dermatological autoimmune disease, and at least one other autoimmune disease, for example, SLE. It is rare globally, especially in African populations. In the two cases, there was no evolution of a new autoimmune disease during their follow-up. Unfortunately, the first case was lost to follow-up in 2010 before reappearing in 2022, where she was last seen. There was no evidence of major organ involvement during her visits. The second case is still being actively monitored; the patient is stable with well-controlled disease. Loss to follow-up is common in low-resource settings mainly due to low socioeconomic status and poor insurance coverage.

In conclusion, there is increasing evidence of the association between thyroid autoimmunity and SLE, which could be explained by the shared demographic, genetic, and autoimmune features. Therefore, every patient with autoimmune thyroiditis, especially HT, should be followed up for possible evolution of SLE. Conversely, patients with SLE with major organ involvement and positive titres of anti-dsDNA and anti-Smith may benefit from thyroid function tests and thyroid antibodies to screen for clinical and subclinical autoimmune thyroid disease. This may help to optimise the outcomes of the treatment of both conditions.

## Figures and Tables

**Table 1:** Laboratory features in the 67-year-old woman.

Haematogram	Reference range	At presentation (2010)	March 2022
Haematocrit (%)	36–48	24.6	36.6
Platelet count (cells/mL)	150,000–400,000	92,000	222,000
ESR (mm/h)	0–20	131	20
Thyroid function tests			
TSH (mIU/mL)	0.27–4.2	8.01	1.22
FT3 (pmol/L)	3.1–6.8	2.5	3.7
FT4 (pmol/L)	12.0–22.0	2.7	8.0
TPOAb (IU/mL)	<5.61	55.5	
Anti-thyroglobulin (IU/mL)	0–10	197.3	
Fasting lipid profile			
Total cholesterol (mmol/L)	<5.12	6.8	
Triglycerides (mmol/L)	<1.17	3.3	
Low-density lipoprotein (mmol/L)	<2.56	4.2	
High-density lipoprotein (mmol/L)	>1.5	0.8	

*Note:* FT3: free tri-iodothyronine, FT4: thyroxine, TPOAb: anti-thyroid peroxidase antibody.

Abbreviations: ESR, erythrocyte sedimentation rate; TSH, thyroid-stimulating hormone.

**Table 2:** Laboratory features in the 23-year-old lady.

Laboratory parameters	Reference range	July 2023	September 2023	August 2025
TSH (μIU/mL)	0.27–4.2	74.29	1.18	1.9
FT3 (pmol/L)	3.1–6.8	3.74	6.01	4.2
FT4 (pmol/L)	12.0–22.0	3.46	21.6	16.8
TPOAb (IU/mL)	<5.61	>2000	—	—
Haematocrit (%)	36–48	28	36	33
White blood cell count (cells/μL)	4000 – 11000	2700	7100	3600
ESR (mm/h)	0–20	114	52	40
24-h urinary protein (grams)	<0.1	5.5	0.3	0.3
ANA	1:<80	1:2560	—	—
Anti-dsDNA (U/mL)	<100	416.87	<10	43.76
Anti-Sm (U/mL)	0–15	>200	—	—
Total cholesterol (mmol/L)	<5.12	5.44	—	3.16
Triglycerides (mmol/L)	<1.17	1.4	—	0.51
Low-density lipoprotein (mmol/L)	<2.56	3.7	—	1.84
High-density lipoprotein (mmol/L)	>1.5	1.08	—	1.09

*Note:* FT3: free tri-iodothyronine, FT4: thyroxine, TPOAb: anti-thyroid peroxidase antibody.

Abbreviations: ESR, erythrocyte sedimentation rate; TSH, thyroid-stimulating hormone.

## Data Availability

Data will be available upon reasonable request to the corresponding author.
